# Tailoring the Performance Profiles of Polycarbonate-Based Engineering Blends Through Formulation and Compatibilization

**DOI:** 10.3390/polym18192339

**Published:** 2026-09-25

**Authors:** Ruogu Tang

**Affiliations:** Department of Animal & Food Sciences, College of Agriculture & Natural Resources, University of Delaware, Newark, DE 19713, USA; rgtang@udel.edu

**Keywords:** polycarbonate, engineering polymer blends, ABS, ABS-g-MAH, compatibilization, phase morphology, formulation-property relationships

## Abstract

Polycarbonate (PC)-based engineering blends were investigated to determine how formulation design and compatibilizer addition influence their mechanical, thermal, thermomechanical, and morphological characteristics. The study was conducted in three stages. First, PC was blended separately with acrylonitrile–butadiene–styrene (ABS), acrylonitrile–styrene–acrylate (ASA), and styrene–butadiene–styrene (SBS) over a range of compositions to compare their effects on mechanical performance. A representative PC/ASA formulation was then selected to evaluate the effect of maleic anhydride-grafted ABS (ABS-g-MAH). Finally, poly(methyl methacrylate) (PMMA) was incorporated into the selected PC/ASA formulation to form a ternary PC/ASA/PMMA system, in which the effect of ABS-g-MAH was further examined. Component identity and loading produced distinct mechanical trade-offs: ABS and ASA progressively reduced strength, stiffness, and elongation at break, whereas SBS better preserved tensile deformation capacity and flexural strength despite reduced stiffness. ABS-g-MAH substantially enhanced ductility and impact resistance in both selected systems, while its effects on stiffness, dynamic mechanical behavior, and thermal properties depended on blend composition and loading. Selective-etching scanning electron microscopy further revealed finer and more uniformly distributed phase morphologies after ABS-g-MAH addition. Overall, the results demonstrate that component selection establishes the underlying performance profile of PC-based blends, while compatibilizer addition provides a further means of adjusting morphology and property balance in a formulation-dependent manner. These findings provide a practical framework for tailoring PC-based engineering blends toward different performance requirements.

## 1. Introduction

Polycarbonate (PC) is an important engineering thermoplastic because of its high impact resistance, dimensional stability, heat resistance, and favorable mechanical properties [1,2]. However, the requirements of engineering applications often extend beyond the property envelope of a single polymer. Polymer blending offers a practical means of addressing this limitation by combining PC with components possessing complementary characteristics [3,4]. Through appropriate formulation, the performance of PC-based materials can therefore be adjusted for different engineering requirements.

A range of thermoplastic and elastomeric components have been incorporated into PC-based blends to modify their mechanical and thermomechanical performance. ABS is one of the most extensively studied blend partners for PC because its multiphase structure can alter the balance between stiffness, strength, and toughness in PC-rich formulations [3,4,5]. ASA provides a related styrenic multiphase architecture but differs from ABS in the nature of its rubbery phase, leading to distinct composition-dependent mechanical responses in PC-based blends [6]. Recent studies on PC/ASA systems have further demonstrated that their mechanical and thermal responses are strongly dependent on formulation and phase structure, reinforcing the importance of composition-specific evaluation of ASA-containing PC blends [7]. SBS, by contrast, acts as a more elastomeric modifying component and can promote different deformation and toughness responses when incorporated into PC-containing systems [8], with previous studies demonstrating composition-dependent changes in mechanical performance in SBS-modified PC blends. PMMA represents a comparatively rigid thermoplastic component and has been employed in multicomponent PC-based blends to adjust stiffness, thermomechanical behavior, and other formulation-dependent properties [9]. Studies of PC/PMMA and related PMMA-containing PC systems have also shown that PMMA incorporation can influence phase morphology, miscibility, and mechanical and thermomechanical behavior [10,11,12]. Although these polymers have individually been studied as blend components or modifiers in PC-containing systems, their effects depend strongly on composition, phase structure, and the particular property being considered. Their inclusion therefore provides a useful basis for comparing how different classes of blend partners redistribute the mechanical performance profile of PC-based materials.

The effectiveness of compositional modification in PC-based blends also depends on the multiphase structure developed during melt blending. Because many polymer combinations are thermodynamically immiscible or only partially compatible, phase morphology, dispersion, and interactions between constituent phases can strongly influence the resulting mechanical and thermomechanical behavior [13,14]. Compatibilization is therefore commonly employed to modify phase organization and improve the overall balance of properties in multiphase polymer blends. Maleic anhydride-functionalized polymers have been widely used for this purpose, and ABS-g-MAH has previously been applied in PC-containing blends to modify morphology and mechanical response [15,16,17]. In the present study, PC/ASA was selected as a representative formulation for evaluating this additional compatibilization variable after the initial comparison of PC blends containing ABS, ASA, and SBS. ABS-g-MAH was subsequently introduced into both PC/ASA and PMMA-containing PC/ASA formulations to determine whether its effects on morphology and material performance depended on the surrounding blend composition.

Although individual PC-based blend systems and compatibilization strategies have been widely investigated, previous studies have often focused on a specific polymer combination, a limited composition range, or a selected subset of properties [18,19]. As a result, direct comparison of how different blend-partner identities, compositions, and compatibilizer additions redistribute the overall mechanical, thermal, thermomechanical, and morphological performance of PC-based blends remains limited. A comparative framework that evaluates these formulation variables under a common experimental design can therefore provide a more systematic understanding of formulation–property relationships and the trade-offs among different dimensions of material performance.

The present study was therefore designed as a three-stage comparative investigation. First, PC blends containing ABS, ASA, and SBS over a range of compositions were evaluated to determine how blend-partner identity and loading influence the mechanical performance profile. Second, a representative PC/ASA formulation was selected to assess the effect of ABS-g-MAH addition on mechanical, dynamic mechanical, thermal, and morphological behavior. Third, PMMA was incorporated into the selected PC/ASA formulation to form a multicomponent system, allowing the influence of ABS-g-MAH to be examined in a different formulation context. Through this approach, the study aims to clarify formulation–property relationships and to determine how compatibilizer addition can further modify the performance balance of PC-based engineering blends.

## 2. Materials and Methods

### 2.1. Materials

Polycarbonate (PC, PC-110), was obtained from Chi Mei Wonderlite (Taipei, Taiwan). Acrylonitrile–butadiene–styrene (ABS), acrylonitrile–styrene–acrylate (ASA), and styrene–butadiene–styrene (SBS), all in general-purpose grades, were supplied by LG Corporation (Seoul, Republic of Korea). Poly(methyl methacrylate) (PMMA; IF850) was also obtained from LG Corporation. Maleic anhydride-grafted ABS (ABS-g-MAH; commercial grade, grafting degree: 2.5%) was supplied by Shenyang Four-Dimensional Polymer Plastic Co., Ltd. (Zhongshan, China). The melt-flow and Molecular weight characteristics of the polymers are summarized in Table 1.

### 2.2. Blend Formulations and Experimental Design

The experimental design comprised three formulation series examining the effects of blend-partner identity, blend composition, and compatibilization on the properties of PC-based engineering blends. In Series I, PC was blended separately with ABS, ASA, or SBS at contents of 10, 20, 30, and 40 parts by mass, with neat PC used as the reference.

In Series II, a PC/ASA blend containing 70 parts PC and 30 parts ASA was used as the base formulation for evaluating the effect of ABS-g-MAH compatibilization. ABS-g-MAH was added at 0, 3, 6, 9, and 12 phr relative to the 100 parts of the PC/ASA base blend.

In Series III, 10 parts PMMA were added to the same 100-part PC/ASA base formulation, resulting in a nominal PC/ASA/PMMA composition of 70/30/10 parts by mass. A fixed PMMA content of 10 parts was selected to introduce a distinct ternary formulation context while retaining the PC/ASA base composition as the common reference for evaluating the effect of ABS-g-MAH. Thus, the ternary formulation contained 110 total parts before ABS-g-MAH addition. ABS-g-MAH was again added at 0, 3, 6, 9, and 12 phr relative to the 100 parts of the PC/ASA base blend, rather than relative to the 110-part ternary formulation. The complete formulations are summarized in Table 2.

The PC/ASA (70/30) formulation included in Series I and the 0 phr reference formulation in Series II had the same nominal composition but were independently prepared and evaluated as controls for their respective experimental series. Because Series I and Series II/III were processed under the series-specific conditions described in Section 2.3, comparisons were made within each experimental series. The experiment design and work flow was illustrated as Scheme 1.

### 2.3. Melt Blending and Specimen Preparation

Prior to melt blending, all raw materials and additives were dried at 95 °C for 4 h. The formulations listed in Table 2 were melt compounded using a twin-screw extruder (SHJ-20, Nanjing Giant Machinery Co., Ltd., Nanjing, China) operated at a screw speed of 300 rpm. After extrusion, the extrudates were naturally cooled and pelletized using a granulator. The resulting pellets were dried again at 95 °C for 4 h prior to injection molding.

For Series I, the temperatures of the four extruder zones were set at 225, 230, 235, and 240 °C, respectively, with the die temperature maintained at 240 °C. The pellets were injection molded using a TTI-130F2 injection molding machine (Welltec Machinery Ltd., Anqing, China). The barrel temperatures from zones 1 to 4 were 230–235, 235–240, 240–245, and 245–250 °C, respectively. The nozzle and mold temperatures were 250–255 and 85 °C, respectively. The injection and holding pressures were 10 and 11.5 MPa, respectively, with a holding time of 10 s.

For Series II and III, the temperatures of the four extruder zones were set at 230, 235, 238, and 240 °C, respectively, with the die temperature maintained at 240 °C. The barrel temperatures during injection molding from zones 1 to 4 were 240–250, 245–252, 248–255, and 250–258 °C, respectively. The nozzle and mold temperatures were 260–265 and 85 °C, respectively. The injection and holding pressures were 10 and 10.5 MPa, respectively, with a holding time of 10 s.

### 2.4. Mechanical and Dynamic Mechanical Characterization

Tensile and flexural properties were measured using a universal testing machine (GT-AT-7000M, Gotech Testing Machines Inc., Taichung City, Taiwan). Tensile tests were performed according to GB/T 1040.1-2006 and GB/T 1040.2-2006 [20,21] using Type 1A specimens at a crosshead speed of 50 mm/min. Flexural tests were conducted according to GB/T 9341-2008 [22] at a test speed of 2 mm/min. Shore D hardness was measured using an LX-D durometer (Shanghai Liuling Instrument Factory, Shanghai, China) according to GB/T 2411-2008 [23], with readings recorded after 15 s. Notched Charpy impact strength was measured using a pendulum impact tester (GT-7045-MDH, Gotech Testing Machines Inc., Taiwan) according to GB/T 1043.1-2008 [24]. Type 1 specimens with a Type A notch were tested edgewise using a pendulum energy of 7.5 J. All mechanical measurements were performed in triplicate.

Dynamic mechanical analysis (DMA) was performed using an EPLexor 500N dynamic mechanical thermal spectrometer (GABO, Niederwinkling, Germany) in dual-cantilever mode. Measurements were conducted from 30 to 140 °C at a heating rate of 3 °C/min, a frequency of 5 Hz, and a dynamic force of 5 N. The damping factor (tanδ) was calculated from the measured loss and storage moduli according to tanδ = E″/E′ at each temperature point.

### 2.5. Thermal Properties Characterization

Differential scanning calorimetry (DSC) was performed using a DSC 204 differential scanning calorimeter (NETZSCH, Selb, Germany) under an argon atmosphere. Samples were first heated from 0 to 300 °C at a heating rate of 20 °C/min, held isothermally at 300 °C for 5 min, and subsequently cooled to 25 °C at a cooling rate of 20 °C/min. Two additional heating-cooling cycles were then performed between 25 and 300 °C using the same heating and cooling rates and the same 5 min isothermal hold at 300 °C. The second-heating curves were used for analysis.

Thermogravimetric analysis (TGA) was conducted using a TG 209F1 thermogravimetric analyzer (NETZSCH, Germany) under an argon atmosphere. Samples were heated from 25 to 650 °C at a heating rate of 10 °C/min.

Vicat softening temperature (VST) was measured using a ZWK1302-B tester (MTS Systems Corporation, Beijing, China) according to GB/T 1633-2000 [25] under an applied load of 10 N and at a heating rate of 120 °C/h.

### 2.6. Morphological Characterization

The phase morphology of the PC-based blends was examined by scanning electron microscopy (SEM) using a JSM-6700F microscope (JEOL, Tokyo, Japan). Fracture surfaces obtained after tensile testing were selectively etched with a 75 vol% tetrahydrofuran (THF) solution in water for 60 s at room temperature to preferentially remove THF-soluble domains and enhance visualization of the phase morphology. After etching, the specimens were vacuum-dried at 45 °C and sputter-coated with a thin layer of gold-palladium (Au/Pd) prior to SEM observation. Micrographs were acquired at an accelerating voltage of 15 kV.

### 2.7. Statistical Analysis

Mechanical property data are presented as mean ± standard deviation (*n* = 3). Statistical analyses were performed separately within each experimental series to evaluate formulation-dependent differences in the measured mechanical properties. One-way analysis of variance (ANOVA) was used to test whether the formulation factor produced a statistically significant overall effect on each mechanical response. When a significant ANOVA result was obtained, Tukey’s honestly significant difference (HSD) multiple-comparison test was applied to identify pairwise differences among formulation means. Differences were considered statistically significant at *p* < 0.05. In tabulated results, different superscript letters within the same property column indicate statistically significant differences among formulation means according to Tukey’s HSD test.

## 3. Results and Discussion

### 3.1. Formulation-Dependent Mechanical Performance of PC-Based Blends

The mechanical properties of the PC-based blends varied with both the type and content of the incorporated polymeric component (Figure 1; Table 3). Increasing the contents of ABS, ASA, and SBS was generally associated with reductions in tensile and flexural strengths, tensile and flexural moduli, and Shore D hardness, although the magnitude of these changes depended on both polymer type and the property considered. More distinct polymer-dependent behavior was observed for elongation at break, whereas notched impact strength varied within a comparatively narrow range across the investigated formulations. Overall, the results show that incorporation of ABS, ASA, and SBS redistributed different dimensions of mechanical performance to different extents, producing composition-dependent trade-offs among strength, stiffness, deformation capacity, impact resistance, and hardness.

The strength and stiffness responses further revealed differences in property retention among the three blend systems. With increasing secondary-polymer content, tensile and flexural strengths generally decreased, but the extent of these reductions depended on polymer type. At a PC/polymer weight ratio of 60/40, tensile strength decreased from 64.4 MPa for neat PC to 49.7, 51.2, and 54.1 MPa for the PC/ABS, PC/ASA, and PC/SBS blends, respectively, corresponding to reductions of approximately 23%, 20%, and 16%. The corresponding flexural strengths decreased from 73.1 MPa for neat PC to 66.2, 65.5, and 70.4 MPa, respectively. Across the composition range, SBS-containing blends therefore generally showed better retention of tensile and flexural strengths than the corresponding ABS- and ASA-containing blends, particularly at intermediate-to-high polymer contents. This pattern, however, was not directly mirrored by the stiffness response. Tensile and flexural moduli also decreased with increasing secondary-polymer content, but their relative differences among the three blend systems were less consistent with those observed for strength. At the 60/40 composition, for example, the tensile modulus of PC/SBS (1.99 GPa) was lower than those of PC/ABS (2.03 GPa) and PC/ASA (2.11 GPa), despite its higher tensile strength. This decoupling reflects the fact that elastic modulus and strength describe different aspects of mechanical response: modulus is governed primarily by the small-strain stiffness of the constituent phases and their relative fractions, whereas strength is additionally affected by stress redistribution, local yielding, and damage initiation within the multiphase structure. Accordingly, strength retention and elastic stiffness did not evolve in parallel with blend composition. Similar property-dependent responses have been reported for PC-based multiphase blends, where mechanical behavior is influenced by composition, phase morphology, and interactions between constituent phases [26,27].

The deformation response showed a more pronounced dependence on polymer type. The elongation at break of neat PC was 56.7% and progressively decreased with increasing ABS and ASA contents, reaching 47.9% and 47.3%, respectively, at a PC/polymer weight ratio of 60/40, corresponding to decreases of approximately 16% and 17%. In contrast, the PC/SBS blends maintained elongation at break within a narrow range of 56.4–58.1% across all investigated compositions, with no statistically significant difference from neat PC. This behavior is consistent with the more elastomeric character of SBS, which can accommodate greater local deformation within the multiphase blend and thereby help preserve tensile deformation capacity even as elastic stiffness decreases. Notably, these differences in elongation at break were not accompanied by comparably large changes in notched impact strength. Although statistically significant differences were observed among some formulations, the mean impact strengths remained within a relatively narrow range of 25.4–27.5 kJ m^−2^ across the entire series. The comparatively modest impact response, despite the distinct changes in tensile elongation, indicates that quasi-static tensile deformation capacity and notch-controlled impact resistance did not evolve in parallel. This distinction is consistent with the different deformation and fracture processes probed under slow tensile loading and rapid notch-controlled impact loading [6,8].

Taken together, ABS and ASA produced broadly comparable composition-dependent responses, whereas SBS exhibited a distinct balance of mechanical properties. Although SBS addition also reduced stiffness and, at higher loadings, tensile strength, it better preserved tensile deformation capacity and flexural strength over the investigated composition range. No single polymer or composition therefore provided superior performance across all measured mechanical properties. Rather, polymer identity and composition determined which aspects of the mechanical response were retained or compromised, emphasizing the importance of evaluating PC-based blends according to their overall performance profiles rather than any individual metric. A nominal PC/ASA weight ratio of 70/30 was subsequently selected as a representative intermediate composition for the independently prepared compatibilization series.

### 3.2. ABS-g-MAH-Mediated Tuning of Mechanical Performance

The tensile and flexural strengths of both PC/ASA and PC/ASA/PMMA blends exhibited loading-dependent responses to ABS-g-MAH addition (Figure 2a,d). For PC/ASA, tensile strength changed little at 3–6 phr but increased at higher loadings, reaching approximately 56 MPa at 9–12 phr compared with 53.4 MPa for the uncompatibilized blend, corresponding to an increase of approximately 5%. In PC/ASA/PMMA, the increase was more progressive, with tensile strength rising from 60.6 MPa at 0 phr to 62.8 MPa at 6 phr and 65.2–66.5 MPa at 9–12 phr, representing an overall increase of approximately 10% at 12 phr. The flexural response followed a different pattern. In PC/ASA, flexural strength increased from 68.1 MPa at 0 phr to approximately 70.5 MPa at 6–9 phr, but decreased again at 12 phr, approaching the value of the uncompatibilized blend. For PC/ASA/PMMA, flexural strength increased from 70.2 MPa at 0 phr to 73.7–75.5 MPa over 3–9 phr, with a maximum increase of approximately 8% at 9 phr, before decreasing to 73.8 MPa at 12 phr. Overall, the strength response became more evident at intermediate-to-high ABS-g-MAH loadings, with tensile strength approaching a plateau at 9–12 phr and flexural strength showing no further benefit at the highest loading. The different loading dependences of tensile and flexural strength indicate that ABS-g-MAH did not act as a conventional reinforcing phase; rather, its addition modified stress transfer and deformation within the multiphase blends in a formulation- and loading-dependent manner. The plateau or partial decline at the highest loading further suggests that increasing compatibilizer content beyond an intermediate range did not produce proportional additional strength gains. Recent studies on compatibilized PC/ABS systems have likewise shown that maleic anhydride-functionalized modifiers can improve strength-related properties, while the magnitude of the response depends on the compatibilizer chemistry and overall blend formulation [28,29].

The strength gains were not accompanied by a corresponding increase in stiffness or hardness (Figure 2b,e,g). For PC/ASA, the tensile modulus remained within a narrow range of approximately 2.07–2.13 GPa, while the flexural modulus varied only slightly between approximately 1.91 and 1.99 GPa across the investigated ABS-g-MAH contents. In PC/ASA/PMMA, both tensile and flexural moduli remained higher than those of PC/ASA throughout the composition range. The tensile modulus showed a modest overall decrease from 2.31 GPa at 0 phr to 2.23 GPa at 12 phr, corresponding to a reduction of approximately 3%, whereas the flexural modulus decreased from 2.42 GPa in the uncompatibilized blend and then remained within a relatively narrow range of approximately 2.31–2.37 GPa after ABS-g-MAH addition. Shore D hardness showed a clearer overall reduction at higher ABS-g-MAH contents, decreasing from 69 to 64 for PC/ASA and from 77 to 71 for PC/ASA/PMMA between 0 and 12 phr, corresponding to decreases of approximately 7% and 8%, respectively. Thus, the observed strength gains were accompanied by largely maintained or moderately reduced stiffness and a more evident reduction in hardness, rather than by overall stiffening. This decoupling indicates that ABS-g-MAH primarily altered the deformation and stress-transfer behavior of the multiphase blends rather than increasing their intrinsic elastic stiffness. Similar nonparallel changes in strength, stiffness, and toughness have been reported in compatibilized PC-based blends [30,31].

In contrast to the limited changes in stiffness, both elongation at break and notched impact strength increased substantially with ABS-g-MAH addition (Figure 2c,f). For PC/ASA, elongation at break increased from 50.1% at 0 phr to 67.2% at 12 phr, corresponding to an increase of approximately 34%. The response was even more pronounced for PC/ASA/PMMA, where elongation increased from 34.8% to 62.1%, an increase of approximately 78%. Notched impact strength showed a similarly strong loading dependence, increasing from 37.5 to 64.2 kJ/m^2^ for PC/ASA and from 28.7 to 57.3 kJ/m^2^ for PC/ASA/PMMA between 0 and 12 phr, corresponding to increases of approximately 71% and 100%, respectively. In both systems, most of the improvement developed progressively through the intermediate loading range, with smaller additional gains at the highest ABS-g-MAH content. The much larger increases in elongation and impact strength than in modulus indicate that ABS-g-MAH primarily enhanced the ability of the blends to undergo deformation and dissipate mechanical energy rather than increasing elastic stiffness. Such behavior is consistent with changes in phase dispersion and stress redistribution within the multiphase morphology, although the present mechanical data alone do not directly quantify interfacial adhesion. These results identify ductility and impact resistance as the mechanical responses most strongly enhanced by ABS-g-MAH, particularly in the PMMA-containing formulation, which exhibited lower initial values for both properties [32,33].

Taken together, the mechanical responses show that ABS-g-MAH acted primarily as a property-balancing modifier rather than a uniform reinforcing agent. No single ABS-g-MAH loading simultaneously maximized strength, stiffness, ductility, and impact resistance, highlighting the formulation- and property-dependent nature of its effect. The most pronounced improvements were observed in elongation at break and notched impact strength, whereas stiffness remained largely unchanged or decreased slightly and hardness declined at higher ABS-g-MAH contents. This contrast further demonstrates that the principal effect of ABS-g-MAH was to redistribute the mechanical performance profile of the blends rather than to produce uniform reinforcement across all measured properties. The origin of these responses is considered further in conjunction with the morphological and thermomechanical results presented in the following sections.

### 3.3. Thermomechanical and Thermal Behavior

Dynamic mechanical analysis demonstrated that the thermomechanical response of the PC-based blends depended strongly on both blend composition and ABS-g-MAH addition (Figure 3a–c). Neat PC maintained the highest storage modulus over much of the lower-to-intermediate temperature range, whereas PC/ASA showed a more pronounced reduction in stiffness as temperature increased. In the binary PC/ASA system, addition of 6 phr ABS-g-MAH did not increase the dynamic stiffness; instead, both the storage and loss moduli were markedly lower over most of the investigated temperature range. By contrast, in the PMMA-containing ternary system, ABS-g-MAH altered the thermomechanical response, with the 6 phr formulation exhibiting higher storage and loss moduli over the intermediate-temperature region than uncompatibilized PC/ASA/PMMA. Increasing the ABS-g-MAH content to 9 phr further changed the magnitude and temperature dependence of these responses rather than producing a monotonic increase. The loss-modulus profiles also showed that the temperature range of energy dissipation varied among the formulations, with the most pronounced response of neat PC occurring at a higher temperature than those of the blends. The calculated tanδ curves further demonstrated pronounced formulation-dependent differences in damping and relaxation behavior (Figure 3c). The position, magnitude, and shape of the tanδ features varied substantially among the formulations, and some curves did not exhibit a fully resolved relaxation maximum within the investigated temperature range. These results therefore support comparison of the relative viscoelastic responses among formulations but were not used independently to assign glass transition temperatures. Overall, ABS-g-MAH did not exert a universal stiffening effect, but instead modified temperature-dependent stiffness, damping, and energy dissipation in a manner that depended on blend composition and compatibilizer loading. Comparable formulation-dependent viscoelastic responses have been reported in compatibilized PC-based blends, where changes in storage modulus, relaxation behavior, and elevated-temperature stiffness depended on both blend composition and compatibilizer type and formulation context [34].

The second-heating DSC thermograms revealed corresponding formulation-dependent differences in the glass transition behavior of the blends (Figure 3d; Table 4). Neat PC exhibited a relatively well-defined transition region, with a Tg of 152.2 °C, whereas the uncompatibilized PC/ASA blend showed a lower glass transition temperature of 146.3 °C. The uncompatibilized PC/ASA/PMMA blend exhibited two distinguishable glass transition features at 147.4 and 155.8 °C, indicating a more complex segmental relaxation response within the ternary multiphase system. Addition of ABS-g-MAH altered these transition characteristics in both the binary and ternary blends. In PC/ASA, the Tg decreased slightly from 146.3 to 144.8 °C after addition of 6 phr ABS-g-MAH. In the PC/ASA/PMMA system, the 6 phr formulation retained two transition features at 145.5 and 154.1 °C, whereas the 9 phr formulation exhibited only one clearly identifiable transition at 145.2 °C. These changes indicate that the segmental thermal response remained sensitive to both blend composition and ABS-g-MAH loading, while the reduced separation of transition features at higher compatibilizer content is consistent with altered phase-related relaxation behavior. No distinct melting or crystallization peaks were observed within the investigated temperature range; therefore, the DSC analysis was focused on glass transition behavior rather than Tm or Tc. Overall, the DSC results indicate that ABS-g-MAH modified the segmental and phase-related thermal response of the blends, although these changes alone are insufficient to establish complete miscibility [35,36].

Thermal stability and heat resistance were evaluated by thermogravimetric analysis and Vicat softening measurements (Figure 3e,f; Table 5 and Table 6). Neat PC exhibited the highest thermal stability, with a *T*_onset_ of 482.00 °C and a *T*_50_ of 490.66 °C, whereas the uncompatibilized PC/ASA and PC/ASA/PMMA blends showed substantially lower decomposition temperatures, with *T*_onset_ values near 407 °C. Addition of 6 phr ABS-g-MAH slightly improved the thermal stability of PC/ASA, increasing *T*_onset_ from 407.21 to 410.43 °C and T50 from 415.34 to 419.19 °C. A more pronounced effect was observed in the ternary system, where *T*_onset_ increased from 407.49 °C for PC/ASA/PMMA to 412.27 and 425.86 °C at 6 and 9 phr ABS-g-MAH, respectively; *T*_50_ and the residue at 650 °C followed the same increasing trend (Table 5). The corresponding DTG curves further confirmed formulation-dependent differences in the temperature range and intensity of maximum mass loss, with neat PC exhibiting a distinctly higher-temperature decomposition maximum than the blended systems and the ABS-g-MAH-containing formulations showing shifts in peak position and profile (Figure 3f). The residual mass at 650 °C reflects the combined decomposition and char-forming behavior of the blend constituents under the inert atmosphere; therefore, the observed residue differences are interpreted as formulation-dependent thermal-decomposition behavior rather than as evidence of a single constituent-specific mechanism. Vicat softening temperatures showed a related but not identical response. Neat PC had the highest Vicat temperature at 151 °C, while PC/ASA and PC/ASA/PMMA decreased to 147 and 134 °C, respectively. ABS-g-MAH slightly increased the Vicat temperature of PC/ASA to 148 °C and partially recovered that of PC/ASA/PMMA to 137 and 136 °C at 6 and 9 phr, respectively (Table 6). The different loading dependence observed for the TGA and Vicat results indicates that thermal decomposition stability and resistance to thermomechanical softening were affected differently by formulation and compatibilizer loading. Neat PC was included as the matrix reference in the TGA comparison, whereas standalone TGA measurements of ABS, ASA, PMMA, and ABS-g-MAH were not part of the present experimental dataset; accordingly, the decomposition behavior is discussed at the formulation level rather than assigned to individual constituents. Recent studies of compatibilized PC-based blends have likewise shown that changes in compatibilizer content and blend formulation do not necessarily produce proportional changes in thermal decomposition stability, indicating that thermal stability remains strongly dependent on the overall blend formulation [37,38].

### 3.4. Morphological Characteristics of the PC-Based Blends

Selective THF etching revealed clear differences in the phase morphology of the PC/ASA blend following the addition of ABS-g-MAH (Figure 4a,b). The uncompatibilized PC/ASA sample exhibited a relatively open and irregular etched surface, with pronounced depressions and heterogeneous phase-related features. After incorporation of ABS-g-MAH, the etched morphology became finer and more continuous, with fewer large irregular etched regions. These changes suggest that ABS-g-MAH altered the phase organization of the PC/ASA blend and promoted a finer and more uniformly distributed etched morphology [39].

A comparable morphological change was observed in the PC/ASA/PMMA ternary system (Figure 4c,d). The uncompatibilized blend showed a rough and heterogeneous etched surface characterized by relatively large depressions and uneven local features, whereas the ABS-g-MAH-containing sample displayed a finer and more evenly distributed morphology. The reduced prominence of large irregular etched features after compatibilizer addition indicates that ABS-g-MAH also influenced phase distribution in the ternary blend, although the SEM observations alone do not directly quantify interfacial adhesion or compatibility [40,41].

Overall, the SEM observations provide qualitative evidence that ABS-g-MAH modified the morphology of both PC/ASA and PC/ASA/PMMA blends. These changes are consistent with the formulation-dependent mechanical and thermomechanical responses described in Section 3.2 and Section 3.3, indicating an association between morphology modification and the observed property changes. However, the present SEM observations should be interpreted as qualitative evidence of morphology modification rather than as direct evidence of improved interfacial adhesion or compatibility. At the same time, the non-monotonic property responses observed with increasing compatibilizer content indicate that morphological modification alone does not translate directly into uniform improvement across all measured properties.

### 3.5. Formulation–Morphology–Property Relationships and Design Implications

Overall, the results show that the performance of the PC-based blends was governed by the combined effects of blend composition, phase morphology, and ABS-g-MAH addition. These factors influenced different dimensions of mechanical, thermal, and thermomechanical behavior to different extents, and their effects were neither uniformly beneficial nor consistently monotonic. The resulting behavior is therefore better interpreted in terms of formulation-morphology-property relationships and overall performance profiles than through the isolated effect of any single component or additive.

The formulation series further showed that the behavior of the PC-based blends was strongly influenced by the identity and concentration of the secondary polymer phase. Incorporation of ABS, ASA, or SBS altered the balance among strength, stiffness, ductility, impact resistance, and hardness to different extents, indicating that these components did not act as interchangeable modifiers. Reductions in strength and modulus were not necessarily accompanied by proportional changes in deformation or impact behavior, demonstrating that formulation changes redistributed the mechanical performance of the blends rather than shifting all properties uniformly. Composition-dependent differences were also evident in the temperature-dependent response of the blends. Selection of the secondary polymer phase therefore establishes the underlying performance profile and defines the range within which subsequent ABS-g-MAH addition can further adjust the balance of mechanical and thermomechanical behavior.

The effect of ABS-g-MAH further demonstrates that its influence on blend performance depended strongly on both formulation and loading level. Its addition altered the mechanical and thermomechanical responses of the binary PC/ASA and PMMA-containing ternary systems in different ways, indicating that the response to ABS-g-MAH was governed by the overall formulation context rather than by compatibilizer content alone. The morphological observations were consistent with changes in phase organization and dispersion, whereas the mechanical and dynamic mechanical results showed that these morphological changes were not accompanied by uniform improvements across all properties. Instead, the overall response indicated a property-dependent effective compatibilizer range: some properties improved progressively up to the highest ABS-g-MAH loadings, whereas others reached a plateau or showed no further benefit at intermediate-to-high contents. ABS-g-MAH therefore acted as a formulation-dependent property modifier rather than as a universally reinforcing additive, emphasizing that compatibilizer loading should be optimized according to the targeted balance of properties rather than maximized on the basis of a single response.

From an engineering-design perspective, these results indicate that optimization of PC-based multiphase blends requires coordinated consideration of both component selection and compatibilizer addition. The blend components first establish the underlying property profile, after which compatibilizer loading can be adjusted to further tune the balance of mechanical and thermomechanical responses. For example, formulations that better preserve stiffness and strength may be more suitable for dimensionally demanding structural components, whereas formulations showing enhanced ductility and impact resistance may be advantageous for housings, protective components, or other applications requiring improved resistance to mechanical shock. The distinct behaviors of the binary and ternary systems further indicate that the effect of ABS-g-MAH should be evaluated within the context of the specific phase composition and morphology of each formulation. Overall, coordinated control of blend composition and compatibilizer loading provides a practical basis for tailoring the performance of PC-based engineering blends.

## 4. Conclusions

This study investigated the tailoring of PC-based engineering blends through formulation design and ABS-g-MAH addition. Variation in the type and content of ABS, ASA, and SBS produced distinct mechanical responses, demonstrating that the balance among strength, stiffness, ductility, impact resistance, and hardness depended strongly on blend composition. In PC/ASA- and PMMA-containing PC/ASA-based systems, ABS-g-MAH further modified these property balances in a loading- and formulation-dependent manner. Ductility and impact resistance were particularly enhanced, whereas stiffness and hardness showed limited improvement or moderate reductions, indicating that no single compatibilizer loading provided uniformly superior performance across all properties. Dynamic mechanical and thermal analyses further showed formulation-dependent changes in temperature-dependent stiffness, energy dissipation, thermal stability, and softening behavior. SEM observations revealed corresponding changes in phase morphology and were consistent with changes in phase dispersion, without directly establishing improved interfacial adhesion or compatibility. Overall, the results demonstrate that the performance of PC-based blends is governed by the combined effects of component selection, blend composition, and compatibilizer loading. Coordinated control of these variables therefore provides a practical basis for tailoring PC-based engineering blends toward different mechanical, thermal, and thermomechanical performance requirements.

## Figures and Tables

**Scheme 1:**
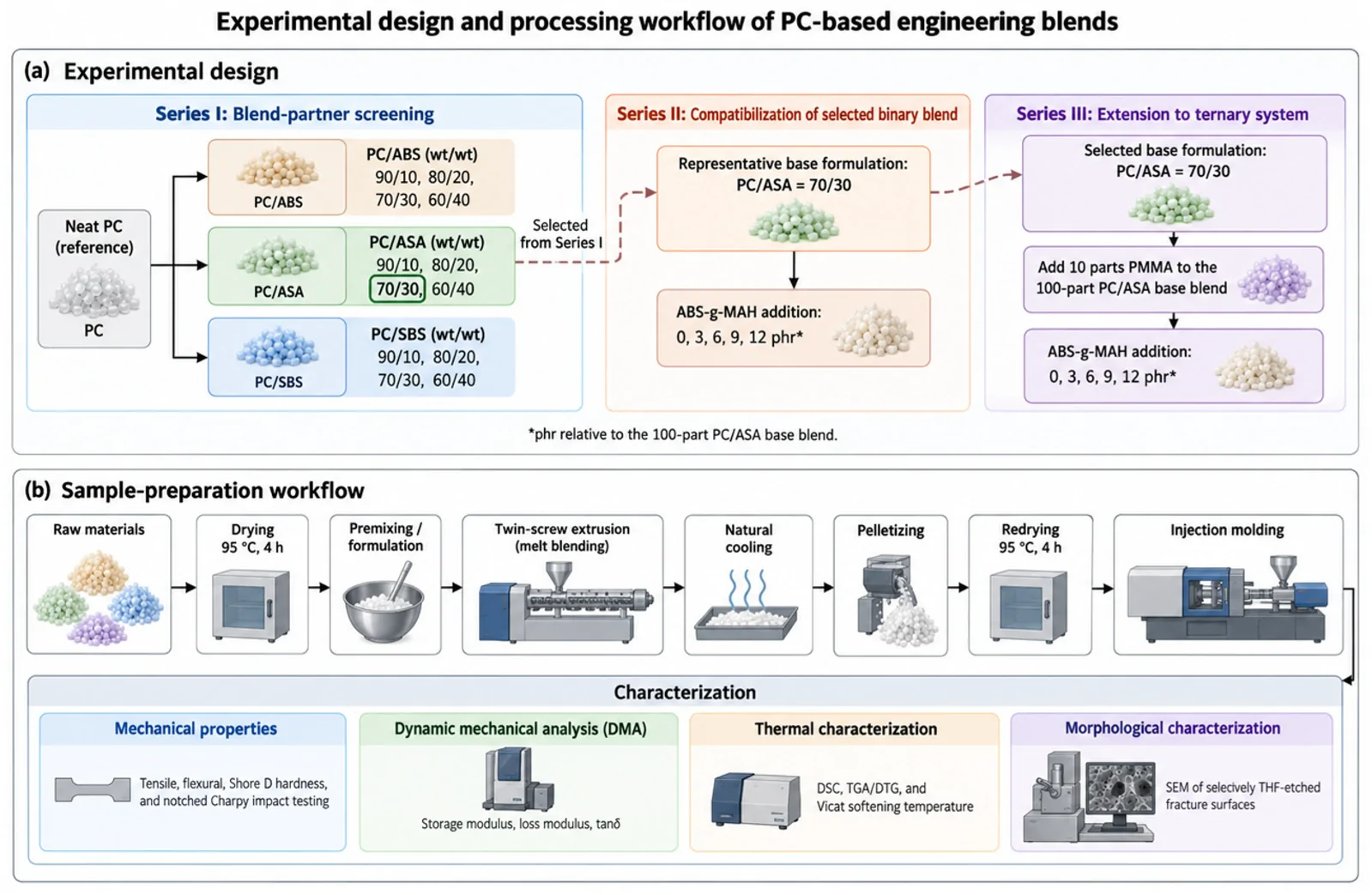
Experimental design and sample-preparation workflow for the PC-based engineering blends investigated in this study.

**Figure 1:**
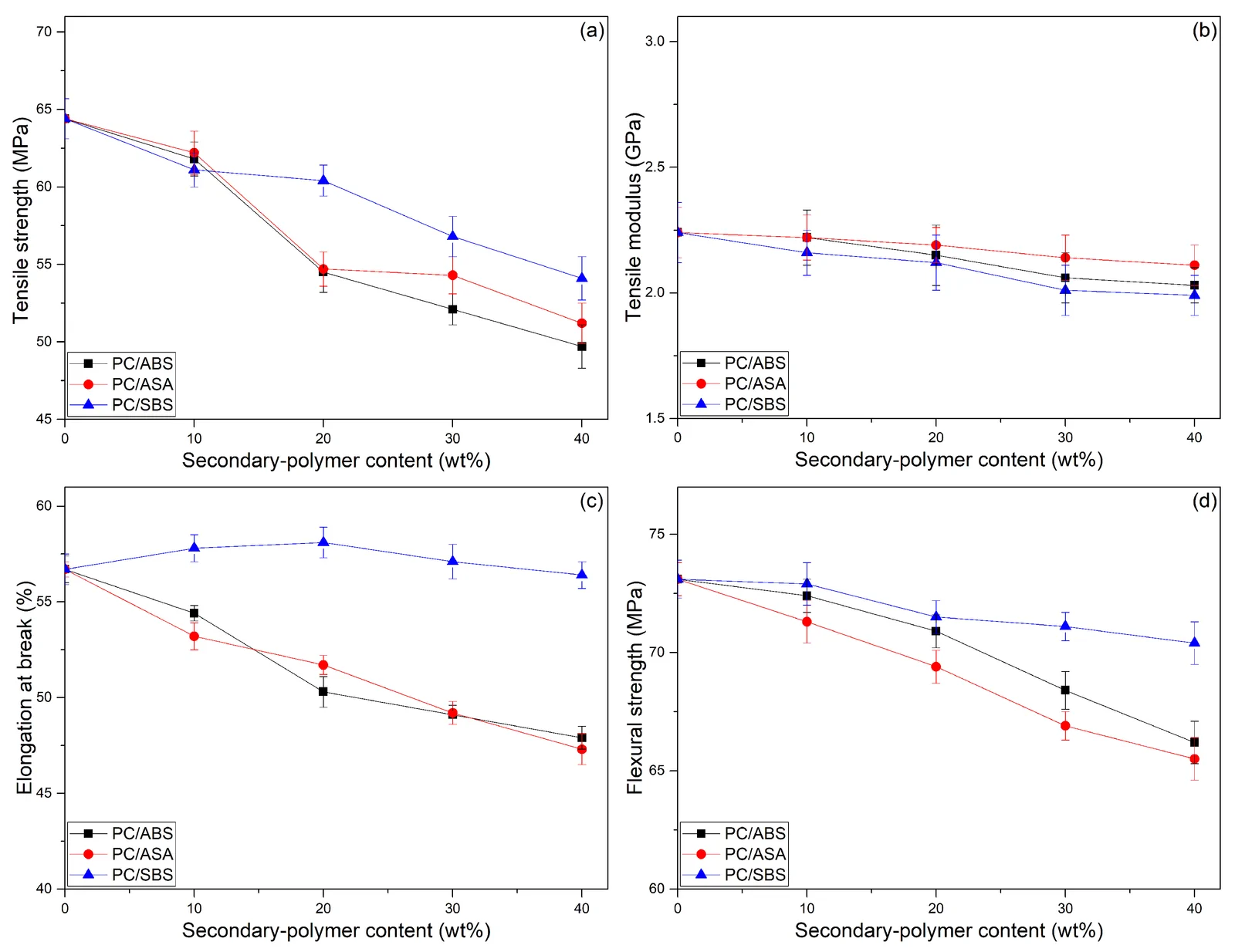

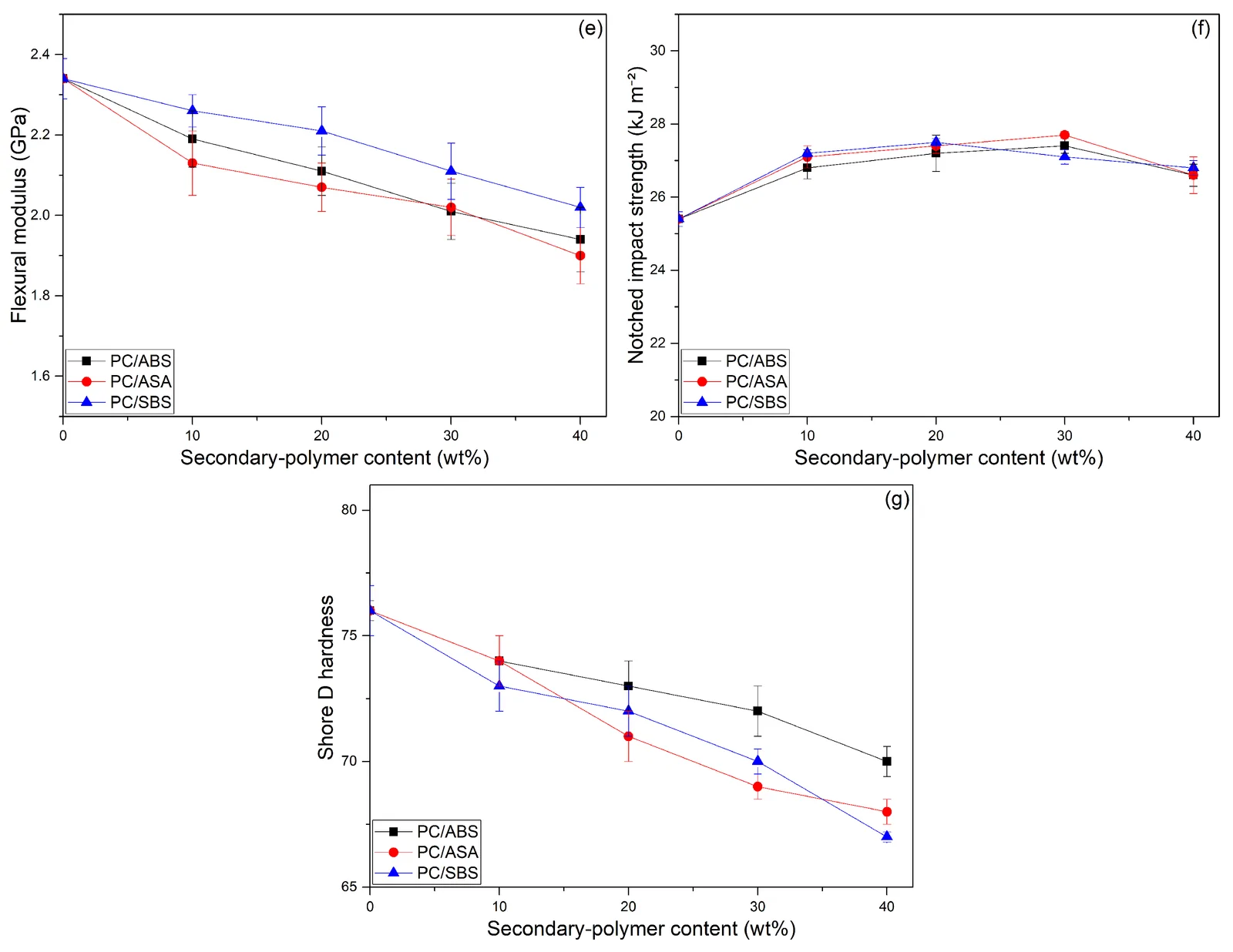
Formulation-dependent mechanical properties of PC-based blends containing ABS, ASA, and SBS at different secondary-polymer contents: (**a**) tensile strength, (**b**) tensile modulus, (**c**) elongation at break, (**d**) flexural strength, (**e**) flexural modulus, (**f**) notched impact strength, and (**g**) Shore D hardness. Data are presented as mean ± standard deviation (*n* = 3). The 0 wt% point corresponds to neat PC and is shared by the three blend series.

**Figure 2:**
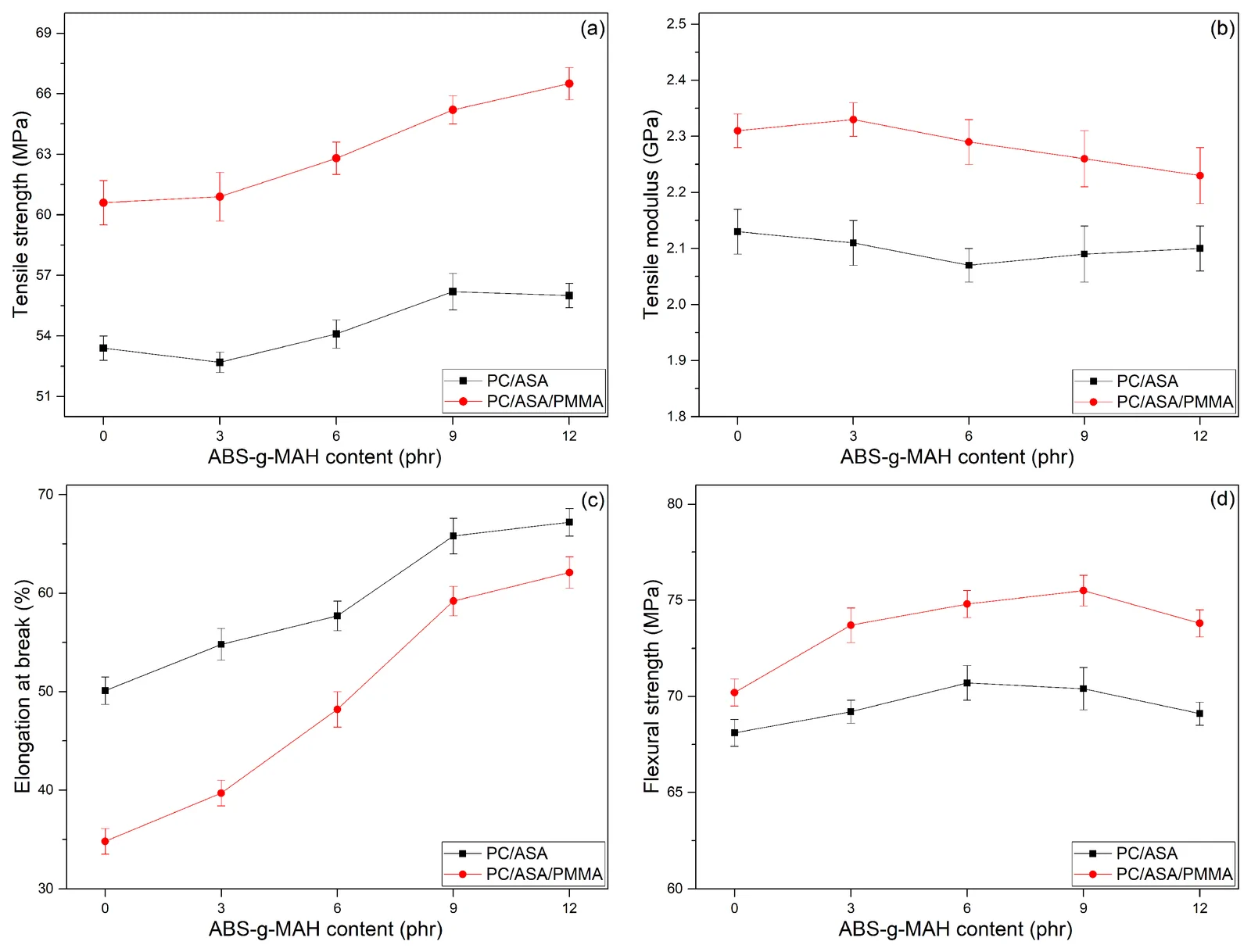

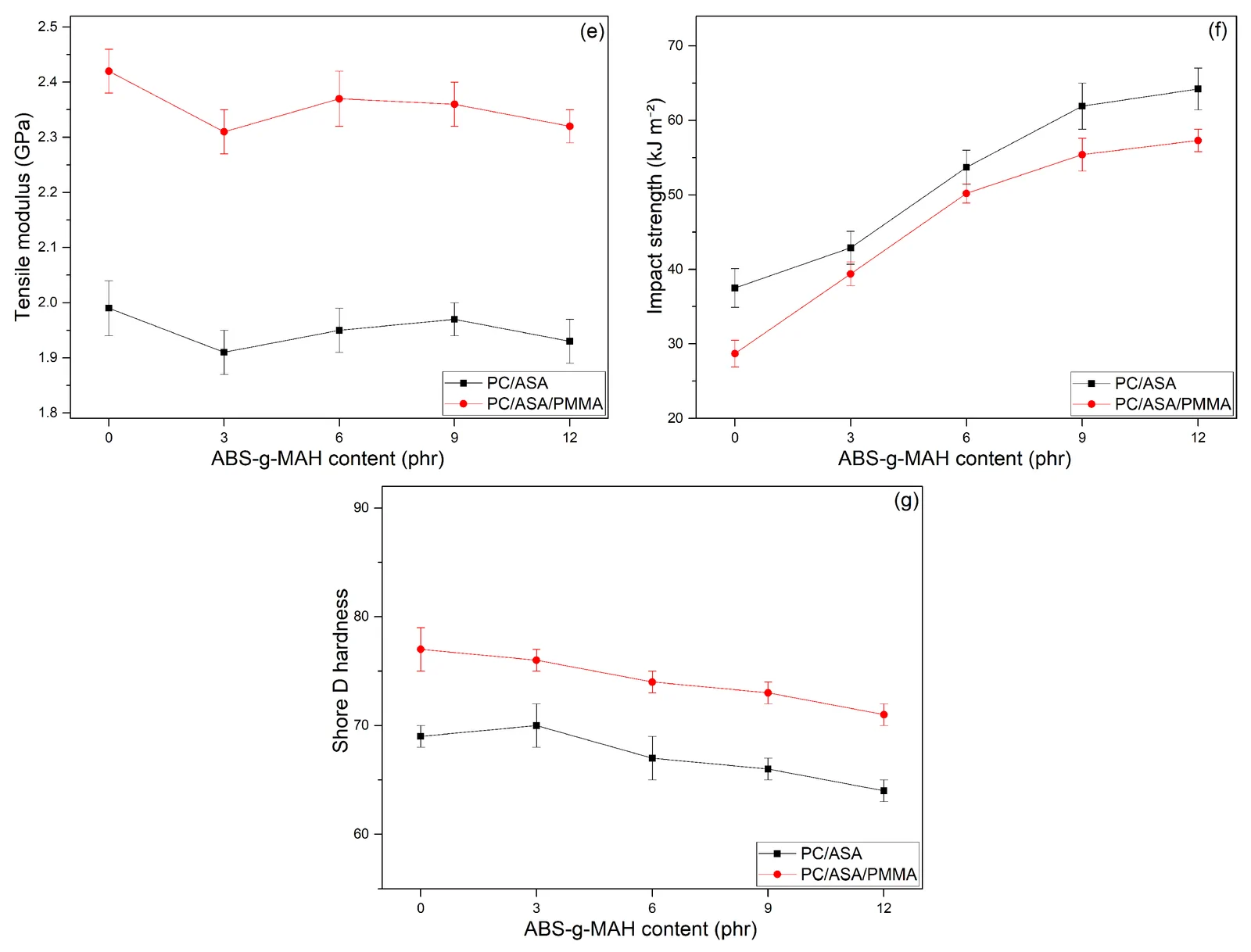
Mechanical properties of PC/ASA (70/30) and PC/ASA/PMMA (70/30/10) blends containing different amounts of ABS-g-MAH: (**a**) tensile strength, (**b**) tensile modulus, (**c**) elongation at break, (**d**) flexural strength, (**e**) flexural modulus, (**f**) notched impact strength, and (**g**) Shore D hardness. Data are presented as mean ± standard deviation (*n* = 3).

**Figure 3:**
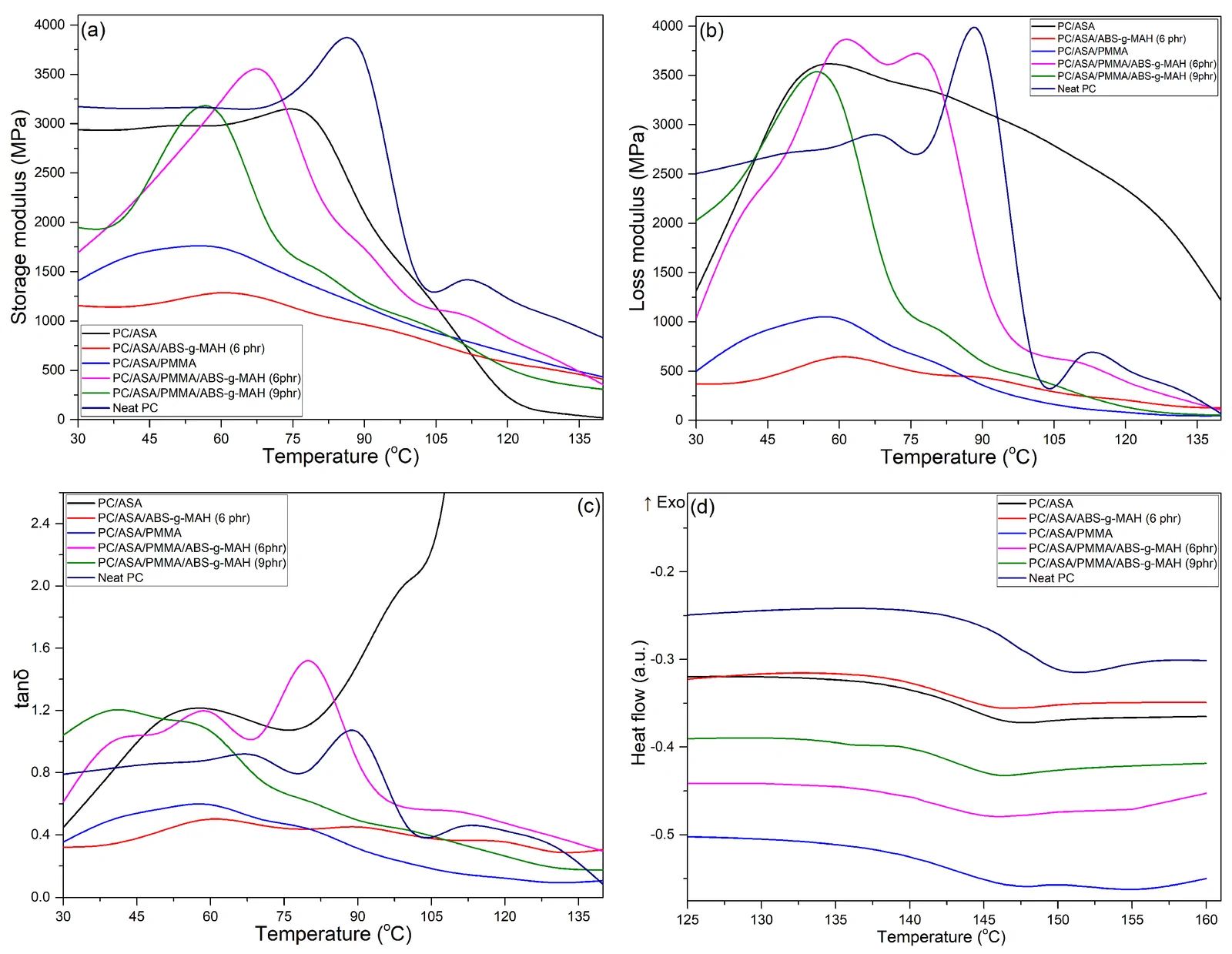

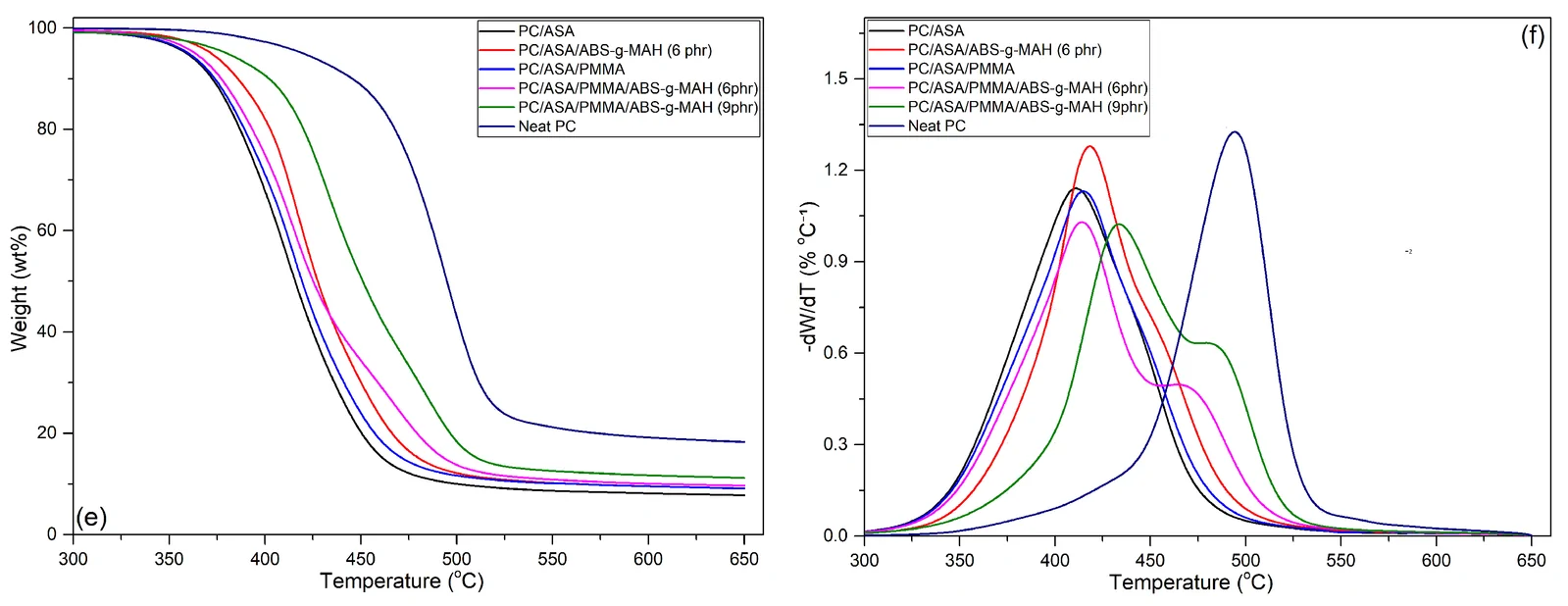
Thermomechanical and thermal behavior of representative PC-based blends. (**a**) Storage modulus (E′), (**b**) loss modulus (E″), and (**c**) damping factor (tanδ) as functions of temperature obtained by dynamic mechanical analysis; (**d**) second-heating DSC thermograms displayed over the glass transition region for clarity; (**e**) thermogravimetric curves; and (**f**) derivative thermogravimetric (DTG) curves. Representative formulations include neat PC, PC/ASA, PC/ASA/ABS-g-MAH (6 phr), PC/ASA/PMMA, PC/ASA/PMMA/ABS-g-MAH (6 phr), and PC/ASA/PMMA/ABS-g-MAH (9 phr). The DSC heat-flow direction is upward.

**Figure 4:**
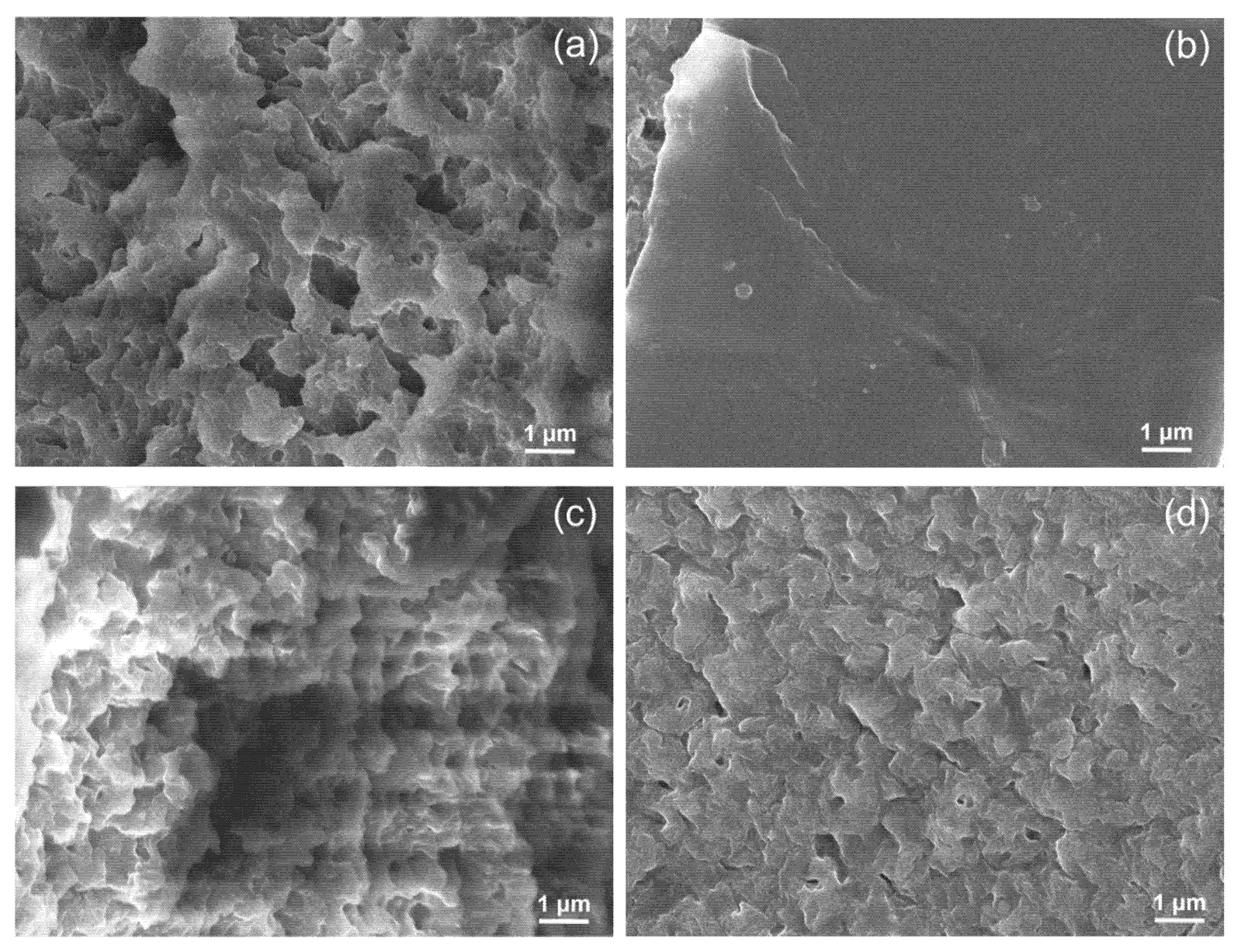
SEM images of THF-etched fracture surfaces of (**a**) PC/ASA, (**b**) PC/ASA with 9 phr ABS-g-MAH, (**c**) PC/ASA/PMMA, and (**d**) PC/ASA/PMMA with 9 phr ABS-g-MAH. All images were obtained at the same magnification (×10,000).

**Table 1:** Melt-flow and Molecular weight characteristics of the polymeric blend components.

Material	MFR (g/10 min)	Mn (g/mol)	Mw (g/mol)	PDI
PC	10	30,000	57,000	1.90
ABS	15	12,000	17,000	1.42
ASA	10	11,500	15,000	1.30
SBS	15	14,000	21,000	1.50
PMMA	13	52,000	85,000	1.63
ABS-g-MAH	—	11,000	18,500	1.68

Note: Mn and Mw denote the number-average and weight-average molecular weights, respectively. PDI was calculated as Mw/Mn. Molecular weight data were obtained from the manufacturers’ specifications.

**Table 2:** Formulations of the PC-based blends.

Series	Blend System	Blend Composition (Parts by Mass)	ABS-g-MAH Addition (phr Relative to the PC/ASA Base Blend)
I	PC/ABS	90/10, 80/20, 70/30, 60/40	—
I	PC/ASA	90/10, 80/20, 70/30, 60/40	—
I	PC/SBS	90/10, 80/20, 70/30, 60/40	—
II	PC/ASA	70/30	0, 3, 6, 9, 12
III	PC/ASA/PMMA	70/30/10	0, 3, 6, 9, 12

Note: For Series III, 10 parts PMMA were added to the 100-part PC/ASA base blend, giving a total of 110 parts before ABS-g-MAH addition. ABS-g-MAH contents are expressed in phr relative to the 100-part PC/ASA base blend.

**Table 3:** Mechanical properties of PC-based blends as a function of blend component and composition.

Formulation (Weight Ratio)	Tensile Strength/MPa	Tensile Modulus/GPa	Elongation at Break/%	Flexural Strength/MPa	Flexural Modulus/GPa	Notched Impact Strength/kJ m^−2^	Shore D
PC	64.4 ± 2.3 ^e^	2.24 ± 0.18 ^e^	56.7 ± 1.3 ^d^	73.1 ± 2.3 ^d^	2.34 ± 0.26 ^e^	25.4 ± 0.4 ^a^	76 ± 1 ^c^
PC/ABS 90/10	61.8 ± 2.1 ^d^	2.22 ± 0.19 ^e^	54.4 ± 1.6 ^c^	72.4 ± 2.5 ^c^	2.19 ± 0.22 ^d^	26.8 ± 0.5 ^b^	74 ± 2 ^b^
PC/ABS 80/20	54.5 ± 1.9 ^c^	2.15 ± 0.12 ^d^	50.3 ± 0.8 ^ab^	70.9 ± 2.1 ^b^	2.11 ± 0.19 ^c^	27.2 ± 0.7 ^bc^	73 ± 2 ^b^
PC/ABS 70/30	52.1 ± 2.3 ^b^	2.06 ± 0.10 ^b^	49.1 ± 1.2 ^ab^	68.4 ± 2.7 ^ab^	2.01 ± 0.13 ^b^	27.4 ± 0.4 ^c^	72 ± 2 ^b^
PC/ABS 60/40	49.7 ± 1.2 ^a^	2.03 ± 0.07 ^b^	47.9 ± 1.1 ^a^	66.2 ± 1.8 ^a^	1.94 ± 0.15 ^a^	26.6 ± 0.5 ^b^	70 ± 1 ^ab^
PC/ASA 90/10	62.2 ± 1.4 ^d^	2.22 ± 0.13 ^e^	53.2 ± 1.4 ^c^	71.3 ± 1.4 ^bc^	2.13 ± 0.12 ^c^	27.1 ± 0.6 ^bc^	74 ± 2 ^b^
PC/ASA 80/20	54.7 ± 1.1 ^c^	2.19 ± 0.14 ^de^	51.7 ± 1.7 ^b^	69.4 ± 1.2 ^ab^	2.07 ± 0.13 ^b^	27.4 ± 0.4 ^c^	71 ± 2 ^ab^
PC/ASA 70/30	54.3 ± 1.3 ^c^	2.14 ± 0.11 ^c^	49.2 ± 1.1 ^ab^	66.9 ± 1.4 ^a^	2.02 ± 0.11 ^b^	27.7 ± 0.3 ^c^	69 ± 2 ^a^
PC/ASA 60/40	51.2 ± 1.2 ^b^	2.11 ± 0.11 ^c^	47.3 ± 1.1 ^a^	65.5 ± 1.1 ^a^	1.90 ± 0.10 ^a^	26.6 ± 0.6 ^b^	68 ± 1 ^a^
PC/SBS 90/10	61.1 ± 2.3 ^d^	2.16 ± 0.13 ^d^	57.8 ± 1.3 ^e^	72.9 ± 1.8 ^cd^	2.26 ± 0.15 ^e^	27.2 ± 0.3 ^bc^	73 ± 2 ^b^
PC/SBS 80/20	60.4 ± 2.0 ^d^	2.12 ± 0.11 ^c^	58.1 ± 1.6 ^e^	71.5 ± 1.9 ^bc^	2.21 ± 0.10 ^d^	27.5 ± 0.3 ^c^	72 ± 2 ^b^
PC/SBS 70/30	56.8 ± 1.7 ^c^	2.01 ± 0.10 ^ab^	57.1 ± 1.2 ^de^	71.1 ± 1.2 ^bc^	2.11 ± 0.11 ^c^	27.1 ± 0.4 ^bc^	70 ± 2 ^ab^
PC/SBS 60/40	54.1 ± 1.4 ^c^	1.99 ± 0.08 ^a^	56.4 ± 1.1 ^d^	70.4 ± 1.3 ^b^	2.02 ± 0.12 ^b^	26.8 ± 0.2 ^b^	67 ± 1 ^a^

Note: Values are presented as mean ± standard deviation (*n* = 3). Different superscript letters within the same column indicate statistically significant differences (*p* < 0.05).

**Table 4:** Glass transition temperatures of representative PC-based blends determined from second-heating DSC curves.

Sample	Tg1 (°C)	Tg2 (°C)
PC	152.2	—
PC/ASA	146.3	—
PC/ASA/ABS-g-MAH (6 phr)	144.8	—
PC/ASA/PMMA	147.4	155.8
PC/ASA/PMMA/ABS-g-MAH (6 phr)	145.5	154.1
PC/ASA/PMMA/ABS-g-MAH (9 phr)	145.2	—

Note: Tg values were determined from the second-heating DSC curves. — indicates that no second glass transition feature was identified.

**Table 5:** Thermal decomposition parameters of representative PC-based blends determined by thermogravimetric analysis.

Sample	Residue at 650 °C(%)	*T*_onset_ (°C)	*T*_50_ (°C)
PC	18.32	482.00	490.66
PC/ASA	7.77	407.21	415.34
PC/ASA/ABS-g-MAH (6 phr)	9.13	410.43	419.19
PC/ASA/PMMA	9.09	407.49	417.27
PC/ASA/PMMA/ABS-g-MAH (6 phr)	9.68	412.27	424.23
PC/ASA/PMMA/ABS-g-MAH (9 phr)	11.21	425.86	438.62

Note: *T*_onset_: onset decomposition temperature; *T_50_*: temperature at 50% mass remaining.

**Table 6:** Vicat softening temperatures of representative PC-based blends.

Sample	Vicat Softening Point (°C)
PC	151
PC/ASA	147
PC/ASA/ABS-g-MAH (6 phr)	148
PC/ASA/PMMA	134
PC/ASA/PMMA/ABS-g-MAH (6 phr)	137
PC/ASA/PMMA/ABS-g-MAH (9 phr)	136

## Data Availability

The data presented in this study within the article. Further inquiries are available on request from the author.
